# Spatial pattern identification (SPI) for ecological modelling

**DOI:** 10.1016/j.mex.2021.101278

**Published:** 2021-02-18

**Authors:** Attila J. Trájer, Viktor Sebestyén, Endre Domokos

**Affiliations:** Sustainability Solutions Research Lab, University of Pannonia, Hungary

**Keywords:** Spatial comparison, Pattern similarity, Key variable identification

## Abstract

There is a growing interest to understand the static and dynamic components of population ranges. In general, the frequently used environmental forecasting and evaluating methods of occurrences like niche-based statistical processes are based on the static evaluation of the causative environmental variables. These techniques do not consider that natural populations of species form the systems of complex, connected networks. The aim of this study was to suggest a possible solution to this methodological problem. The proposed variable pattern comparison tool (*Spatial pattern identification (SPI) for ecological modelling*) provides an opportunity of deep examination of spatial connections between environmental variables and occurrence data in GIS models. The idea of the developed method is, that the network characteristic of the primary point-like occurrence data provides statistically evaluable new and valuable information about the nature and reasons for the interconnections of populations. In technical sense, the approach is based on which the key variables of the models can be identified, thus establishing the targeted variable selection and possible solutions for model reduction.

• Exploring the relationships between variables of a GIS model.
• Static and pattern similarity-based comparison of the model variables.
• Identification of key variables of the model and model reduction.
• The network allows the understanding intra- and interspecific population connections.

## Graphical abstract

**Figure:**
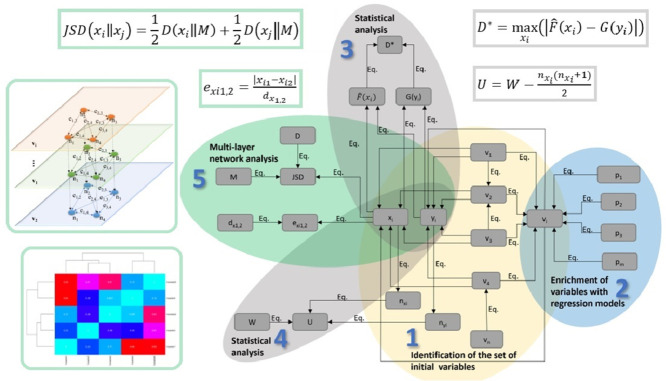

Specifications table

**Table:** 

Subject Area:	Environmental Science
More specific subject area:	*Bioclimatic factors, spatial modelling, network analysis*
Method name:	*Spatial pattern identification (SPI)*
Name and reference of original method:	De Domenico M, Nicosia V, Arenas A, Latora V (2015) Layer aggregation and reducibility of multilayer interconnected networks. Nat Commun 6:6864. https://doi.org/10.1038/ncomms7864
Resource availability:	*NA*

## Method details

**Fig. 1:**
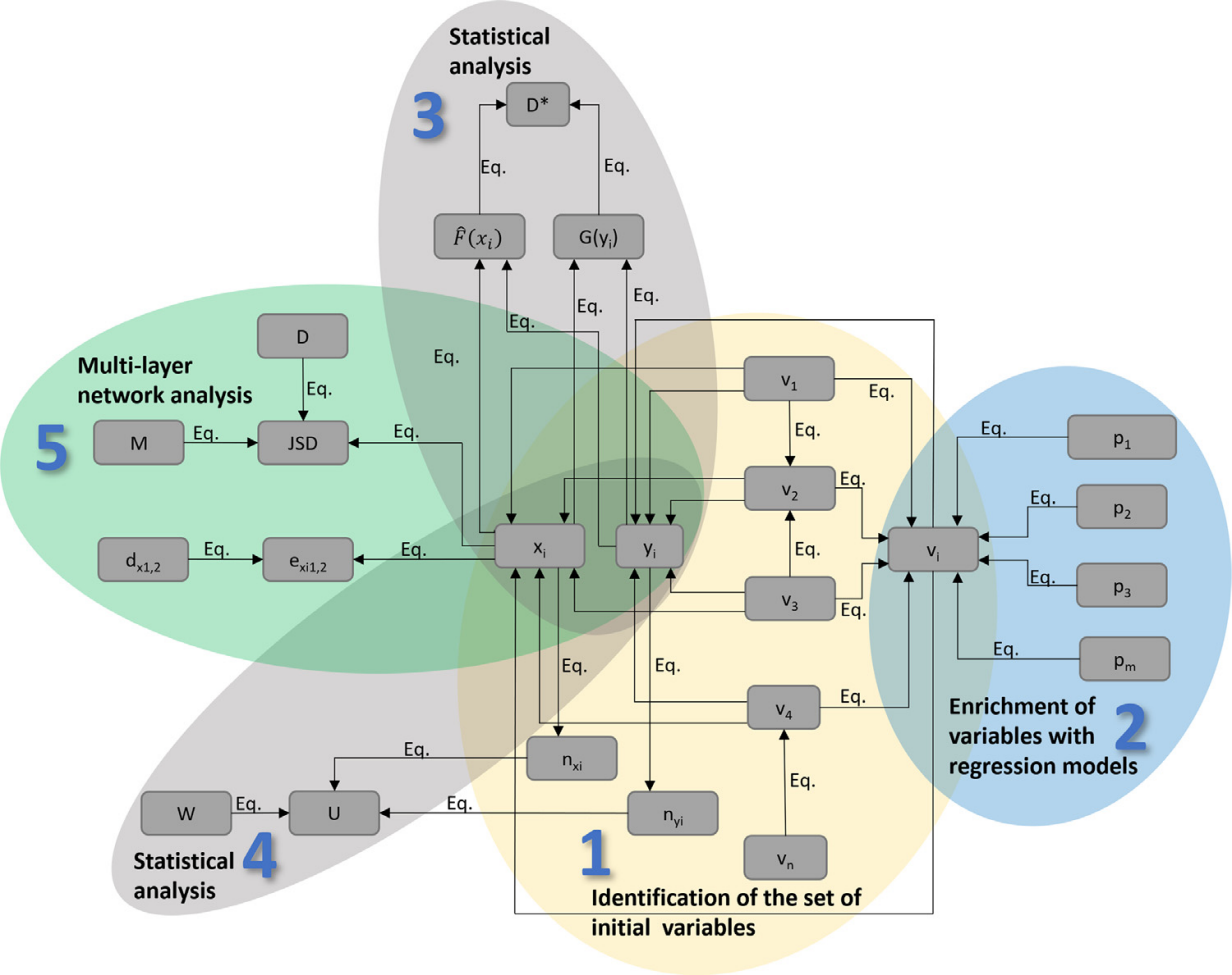
The process of the proposed multilayer network-based comparative variable evaluation (SPI) method.

In the case of environmental problems, differences in samples are key to understanding, which is usually assessed by static comparison of measurement results. The main advantage of the proposed methodological development is that, in addition to static comparison, it also takes into account differences based on the spatial location of samples (similarity of changes in variables), thus providing more information for a better understanding of the constructed models. The purpose of the Spatial pattern identification (SPI) analysis tool is to identify key variables of the problem examined based on geographic environmental models. The proposed exploration tool has been successfully used to explore the links between malaria and human settlements [1]. The steps of the proposed approach are summarized in Fig. 1.

The analysis begins with the pre-selection of geographical variables (e.g., bioclimatic variables) when the analyst selects available indicators related to the problem under study (step 1.). It is important to highlight that the analysis can be enriched by adding external variables (step 2.) that can be fitted to the GIS model using multiple regression models. Next, the multilayer network must be defined, where the nodes are the sampling sites, and the edges are straight lines connecting the sampling sites. The layers of the network are defined based on the examined variables (step 5.). The weight of the edges can be determined by the distance and the difference in the given variable. Static comparison of sampling sites is done by statistical tools (steps 3-4.). However, the results are evaluated together.

To determine the differences between the sites where the given environmental factor is reported and where it is missing (blind sample), two-sided one-sample Kolmogorov-Smirnov tests were used. The Kolmogorov-Smirnov test is a non-parametric test which can verify whether the cumulative distribution function of the selected dataset is the same as the assumed cumulative distribution function. To analyze the full-occurrence model, the function of the test can be given in the following form [2], [3], [4], [5]:

(1) $$D^{*}=\max_{x_{i}}\left(\left|\hat{F}\left(x_{i}\right)-G\left(y_{i}\right)\right|\right)$$

Where $\hat{F}\left(x_{i}\right)$ is the empirical cumulative distribution function of the human settlements in the sampled points of the modelling variables (x_i_) during the period in question and G(y_i_) is the cumulative distribution function of the blind pattern in the sampled points of the modelling variables (y_i_).

**Fig. 2:**
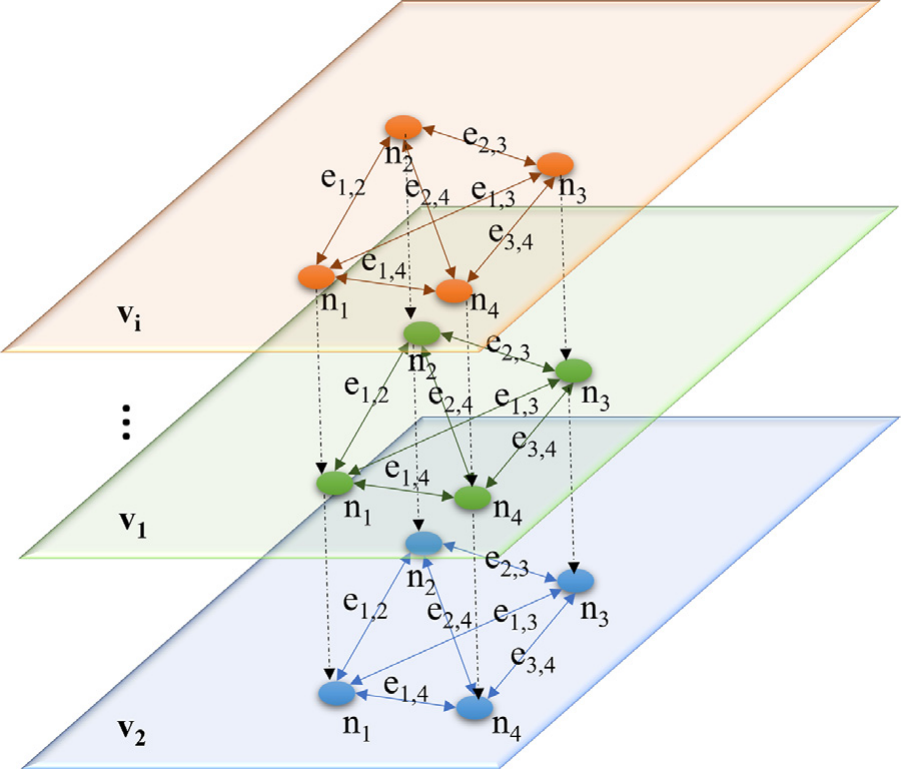
The multilayer network for the SPI method [8].

The Mann-Whitney U-test statistic was performed to test the H0 hypothesis that blind and settlement point sets originate from the same population. The test is related to the Wilcoxon rank-sum statistic in the following way according to the paradigms of Gibbons and Chakraborti [6] and Hollander and Wolfe [7]:

(2) $$U=W-\frac{n_{x_{i}}\left({n_{x}}_{i}+1\right)}{2}$$

where U, is the number of times a x precedes an y in an ordered arrangement of the elements in the two independent samples x_i_ and y_i_. W is the sum of the ranks in sample 1.

(3) $$e_{xi1,2}=\frac{\left|x_{i1}-x_{i2}\right|}{d_{x_{1,2}}}$$

where the e_x1,2_ is the weight of the edge between the given two nodes, x_i_ is the value of the variable at node x_1_, x_2_ is the value of the variable at node x_2_, d_x1,2_ is the distance between the nodes x_i1_ and x_i2_. The logic of parameterizing the multilayer network is shown in Fig. 2.

The multilayer network is defined as shown in Fig. 2. The sampling points are the same for all variables. The sampling points are connected to each other, and then the differences in the given variable are calculated in pairs, which are corrected by the real geographic distance between the sampling points. The multilayer network variables are compared based on the Pairwise Quantum Jensen-Shannon Distance (JSD) [9]:

(4) $$JSD\left(x_{i}∥x_{j}\right)=\frac{1}{2}D\left(x_{i}∥M\right)+\frac{1}{2}D\left(x_{j}∥M\right)$$

**Fig. 3:**
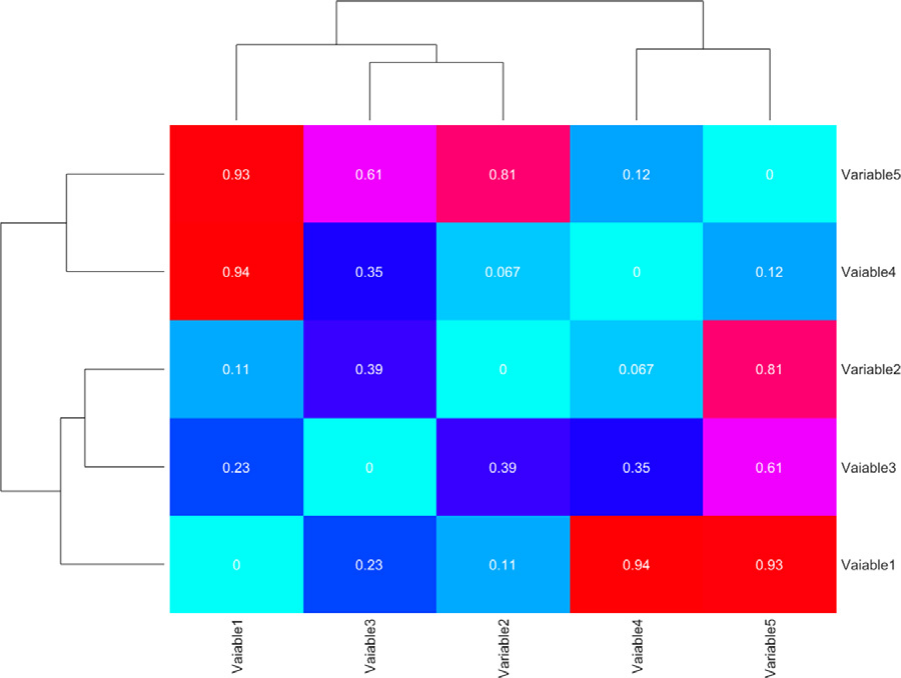
The clustergram of the JSD values.

Where M= ½ ($x_{i}$+$x_{j}$) is the mixture distribution, and D is a symmetrized and smoothed version of the Kullback–Leibler divergence. The relationships between the variables can be visualized based on a cluster diagram, which is illustrated in Fig. 3.

Fig. 3 shows that variables with a similar spatial pattern are given a lower JSD value, while variables with a different spatial pattern are represented with high JSD values. In this example, if Variable1 is the variable to be analyzed, it shows a very close pattern similarity to Variable2, so this variable can be identified as a key variable, while the spatial variation of variables 4 and 5 is different, so further examinations are needed to assess their applicability in the model.

## Applicability

The proposed Spatial pattern identification (SPI) approach is used to assess the variables of GIS models. It is suitable for extending analyzes based on the comparison of commonly used points, considering spatial diversity, which contributes to a higher-level understanding of the relationships describing the problem under study. The SPI tool has been successfully used to identify patterns between malaria incidence and bioclimatic and derived variables, and that can be used to test the theory of human settlement formation in the Middle Paleolithic era in Africa and South Asia [1]. The method can also be used, for e.g., the joint investigation and prediction of the present and the potential future changes of the human populations and disease factors related to the multivariable environmental matrix of ecological factors.

## Declaration of Competing Interest

The authors declare that they have no known competing financial interests or personal relationships that could have appeared to influence the work reported in this paper.
